# What I didn’t grow up with is dangerous: personal experience with a new technology or societal change reduces the belief that it corrupts youth

**DOI:** 10.3389/fpsyg.2023.1017313

**Published:** 2023-10-12

**Authors:** John Protzko, Jonathan W. Schooler

**Affiliations:** ^1^Department of Psychological Science, Central Connecticut State University, New Britain, CT, United States; ^2^Department of Psychological & Brain Sciences, University of California, Santa Barbara, Santa Barbara, CA, United States

**Keywords:** societal decline, digital-technology use, social media, screen time, preregistered, moral panic, generational perceptions, cultural adaptation resistance

## Abstract

**Introduction:**

Throughout history, technological and societal changes consistently receive suspicion. Their influences appear damaging, corrupting, and potential precursors to societal downfall, with today’s youth often portrayed as the primary victims. This study aims to explore an underlying reason for these perceptions and to investigate why society frequently perceives technological and societal transitions as detrimental to the younger generation.

**Methods:**

We conduct two studies across a total of 1,702 participants. In a pilot study, American adults generate a list of technological/societal innovations they believe to be especially problematic for youth in various ways. The second study maps beliefs that specific technological/societal shifts are corruptive, correlating with whether American adults experience them during their upbringing.

**Results:**

People view recent technologies as particularly corrupting of today’s youth. A notable within-person correlation exists between an individual’s exposure to specific technologies during their youth and their belief that these technologies corrupt today’s youth. Specifically, people are more inclined to view technological/societal shifts as corruptive if they don’t experience them during their formative years (*b* = −0.09, *p* < 0.001, 95%CI = [−0.11, −0.09]). When reminded of their own exposure to a particular innovation during their upbringing, however, this relationship reduces.

**Discussion:**

These findings suggest unfamiliarity currently stands as a pivotal factor in societal apprehensions regarding new technological and societal evolutions. As society welcomes new innovations, an enduring cycle emerges where those unacquainted changes seem corruptive to the newer generations. Recognizing this bias, primarily driven by mere unfamiliarity, may be crucial for more balanced evaluations of the inevitable technological and societal progress.

## Introduction

People have been complaining about the corruption of the youth and the decline of society for thousands of years (Smart, 1836; Freeman, 1907). These apparent declines have, throughout history, been blamed on changes in society and technology. Social Media in the early 21st century has been seen as corrupting the youth (e.g., Marwick, 2008 Twenge, 2017), yet concerns about the devastation the radio has on children (e.g., Preston, 1941; Wartella and Jennings, 2000) and the panic about radio’s ability to create a single mass culture with no dissenting opinions (e.g., Davis, 1965; Swingewood, 1977) have faded. Nevertheless, in the early 20th century radio was seen as an invading force that crowded out more intellectual past times such as reading (Eisenberg, 1936; Wartella and Jennings, 2000). Reading, however, was the enemy in the 18th and 19th centuries, leading to the belief that novels led to a frittering away of the young mind (e.g., Hitchcock, 1710; see also Furedi, 2015; Proulx, 2019), while people in the early 21st century complain that the youth do not read enough (Protzko and Schooler, 2019). It appears that we cannot keep our stories straight across generations about *which* technological/societal changes are actually corruptive of the youth. Why do we keep accusing the technologies and societal changes of the day of corrupting the youth?

### Shifting foci

Examples of historical complaints about the corrupting influences of then-present technological/societal changes abound and are entertaining to review (see Orben, 2020, for more examples). Socrates criticized writing as a technology that would degrade the youth as it “will create forgetfulness in the learners’ souls, because they will not use their memories; they will trust to the external written characters and not remember of themselves” (Plato, 360bce/Jowett, 2019). One would be hard pressed to find people complaining about the corrupting influence of books, the printing press, or writing in the present.

There is the persistent and historically pervasive belief that ‘kids these days’ are in decline, dating back to at least the 5th Century bce (e.g., Protzko and Schooler, 2019). People largely view the youth of ‘the present,’ regardless of what present it is, as deficient compared to previous generations (see also Trzesniewski and Donnellan, 2014; Protzko and Schooler, 2022). If the youth of ‘the present’ are seen as in decline, people may want something to blame.

Historically, explanations for the decline of the youth have tended to focus on contemporary technological/societal changes. The focus often seems to be on ‘new’ technological/societal changes, not ones that have been around for generations. Complaints about the printing press, corrupting people with bombardments of information (e.g., Gessner, 1565, as cited in Blair, 2003), were largely limited to when the printing press was a newer societal advance. At the time, complaints were no longer about writing in general (as Socrates bemoaned around 1,200 years earlier) but about the new alleged problems introduced when reading was possible *en masse*.

Numerous reports have mocked these historic complaints, and some have even related such historic concerns to modern panics over contemporary technological/societal advances (see Bell, 2010; Gillard, 2018; see also Smiley and Fisher, 2022), yet the warnings of the new technological/societal change persist (e.g., Twenge et al., 2020). Why the constant panic? We propose a lack of personal experience with the technology/societal change as one psychological reason why adults perennially view new technology/societal change as a source of the decline of the youth of the day.

### Personal experience

Social Media arose and grew in popularity in the 1990s and 2000s, becoming ubiquitous in the 2010s (Pew Research Center, 2021). People born during the 1980s have the experience of growing up without *and with* such technologies, depending on when they adopted the technology. People born in the 2000s–2010s have little to no experience of not living in an internet and social media-connected world. People born before the 1980s have no personal experience growing up with the internet or social media. This lack of personal experience may color people’s understanding of the technology or social change.

The stark differences in the experiences of individuals who grow up with versus without a particular technology raises the following possibility: individuals who lack access to a given technological/societal change while growing up may perceive grave risks of that technology for youth of ‘the present’ who are exposed to it. This would explain why people in the early 20^th^ century, lacking any experience growing up with the radio, would have seen it as such a corrupting influence on youth (e.g., Eisenberg, 1936; Wartella and Jennings, 2000) while it may not be seen as dangerous to people in the early 21st century, as nearly all Americans have the experience of growing up with the radio.

Personal exposure may help explain why we see the next technology/societal change as dangerous to the youth of ‘the present’ while being much less concerned with those that we grew up with ourselves. Of course, one’s generation and their exposure to a technology is often confounded, as one cannot experience a technology that was not available. Nevertheless, the fact that individuals within a generation can vary in experience with particular technologies enables us to potentially unconfound these two variables. Specifically, the personal exposure hypothesis predicts that within a generation those who were exposed to a particular technology should be less inclined to perceive today’s youth as at risk from that particular technology relative to those who had less exposure.

Importantly, we make no claims about the empirical status of *whether* a given technological/societal change is itself harmful (see, Orben and Przybylski, 2019 for effect sizes from correlational work, for example), but rather seek to understand a psychological underpinning of why society’s focus keeps shifting across ‘corrupting influences’. To better understand how exposure to technologies may impact people’s assessments of the challenges facing “kids these days (Protzko and Schooler, 2019 2022) we investigated two questions: (1) In general, are anecdotal reports accurate in suggesting that adults perceive children at greater risk from newer technologies relative to ones that were available to their generation (i.e., is social media perceived as being more dangerous than television)? (2) Are individuals who themselves lacked experience with a specific technology/societal advance especially inclined to perceive that particular technology as problematic?

As people have been rejecting new technologies and innovations since at least Plato’s day (Plato, 360bce/Jowett, 2019) while remaining quieter on the perceived dangers of older technologies, we conduct a systematic study of the possibility that people are more likely to see ‘current’ technologies as dangerous to the youth than older technologies. Assuming this relationship is shown to be true, we then try to understand one explanation for the distrust of ‘current’ technologies—people’s personal experience with the technology. We propose that a lack of experience with a technology or social change leads people to think it is dangerous for the youth of ‘the present’, which leads to the following straightforward prediction: for any given advance those who grew up with it will perceive it as being less problematic than those who did not.

## Methods

We first ran a pilot study to elicit user-generated technological/societal influences that Americans believe are responsible for corrupting the youth. Participants were asked to identify the reasons or causes of the decline of the youth of ‘the present’. Participants were randomly assigned to read one of nine different questions: either a general or a specific question referring to one of eight particular potential forms of decline. Participants reading the general decline question read:

Children and youths today appear noticeably in decline from the standards of youth of the past. We would like to know what you think the reasons or causes of this decline are.

Below are five open spaces. Think about what you think is causing a decline in the youth of today. For each line, please write less than five words. So for example, if you think ‘social media’ is one reason, write that below. Please write whatever you honestly believe are the causes or reasons.

For the specific declines, participants were told that children and youths today appear in decline on one of the following domains: they seem to: *be getting more narcissistic*, *be reading less*, *be more politically extreme*, *be lazier*, *lack the desire to work hard*, *be less respectful of authority*, *be too sensitive and politically correct*, *be more violent*. This list was chosen to reflect commonly heard complaints against the youth (e.g., Trzesniewski and Donnellan, 2014 Protzko and Schooler, 2019 2022) and was not meant to be comprehensive. The specific questions were meant to make the task easier for participants. We collected the responses that all participants gave and reviewed them to try to locate additional, commonly occurring themes. No formal analysis was undertaken.

Participants were 202 members of Amazon Mechanical Turk (mTurk) who were given the survey from November 10, 2019 to November 12, 2019. As there was no confirmatory analysis planned or effect size sought or inferential statistics applied in this pilot study, no power analysis was conducted. See materials and data at https://osf.io/4zk9e/. The responses from this pilot study were used to populate potentially corrupting technologies and societal changes for the main study.

### Main study: does not growing up with a technology increase the belief it is corrupting the youth?

The purpose of this study was to relate exposure to certain technological/societal advances in childhood (identified both historically and in the pilot study) to the belief that those advances are corrupting youth of ‘the present’.

### Methods

#### Materials

We populated our list of technologies and societal advances from historical complaints, contemporary complaints, and user-identified causes from the pilot study. Participants were given the list twice, once when they were asked what they believe contributes to the decline and corruption of the youth, and again when they identified whether they personally had access to any of these technologies or societal advances growing up. All items were presented in random order. The full list of ‘corrupting’ technologies and societal advances can be seen in Table 1, along with the base overall rate at which people believed the influence had a corrupting influence.

**Table 1:** Univariate percentages of American adults who believe each item is specifically corrupting the youth of ‘the present.’

Technology or societal element	% Who believe it is corrupting the youth
Social Media	72.9
Smart phones	53.9
The Internet	58.8
The Radio	5.3
Television	31.0
Reading novels	2.1
Driving cars	4.4
Single parent families	48.6
Video Games in the home	46.9
Heavy Metal music	14.9
24-h news	12.1
Dance clubs	6.5
Nicotine vaporizers	40.5
Netflix	14.1
Jazz music	1.5
Long hair	3.4
Ballroom dancing	1.3
Motion pictures	14.1
Not going to church	37.3
Online dating	22.1
Calculators	2.9
Autocorrect	7.5
Word processors	2.8
Audio/Electronic books	4.3

#### Procedure

Participants were first be asked what year they were born on a drop down list from 2001 until 1918. Next, participants were randomly assigned to fill out the list of what they had growing up or what they believe is causing a decline in the youth scales in random order. For the *What I Had* scale, participants read:

Below is a list of different technologies and aspects of society.

Please select below all of the items that you personally had or experienced growing up.

So for example, if TV was around while you grew up but you never watched it, you would not check Television.

For the *Corruption* scale, participants read:

Children today appear to be in decline compared to the way children were when you were a child.

We are interested in what you believe contributes to this decline. Below is a list of possible causes. Please select as many as you honestly believe are contributing to the decline of the youth of today.

Participants were given the list of items, presented in random order, and allowed to check as many options as they saw fit.

#### Analysis plan

The analysis is a within-person analysis, with the prediction that if someone had exposure to a given technological/societal change growing up, they would be less likely to view it specifically as corrupting the youth. This within-subjects mixed effects model includes whether the individual believes the technological/societal change is corrupting as the binary dependent variable, and whether they had exposure to the technology as the independent variable, run with robust standard errors. The model was not able to converge using the technological/societal change as a random-effect, so it was included as a fixed-effect. Analysis scripts, data, and all materials are available at https://osf.io/4zk9e/. This study was preregistered prior to data collection at https://osf.io/yrzxw.

#### Participants

We collected 1,500 participants, drawn in a stratified way with unequal probabilities of selection, so that the people who complete each survey will resemble the nation’s adult population (according to the most recently available Current Population Survey, conducted by the U.S. Census Bureau) in terms of gender, age, education, ethnicity (Hispanic vs. not), race (allowing each respondent to select more than one race), region, and income. The data was collected in December of 2019. The sample size was determined as this study was part of a project running studies with fixed sample sizes at *N* = 1,500 (Protzko et al., 2020).

## Results

**Figure 1:**
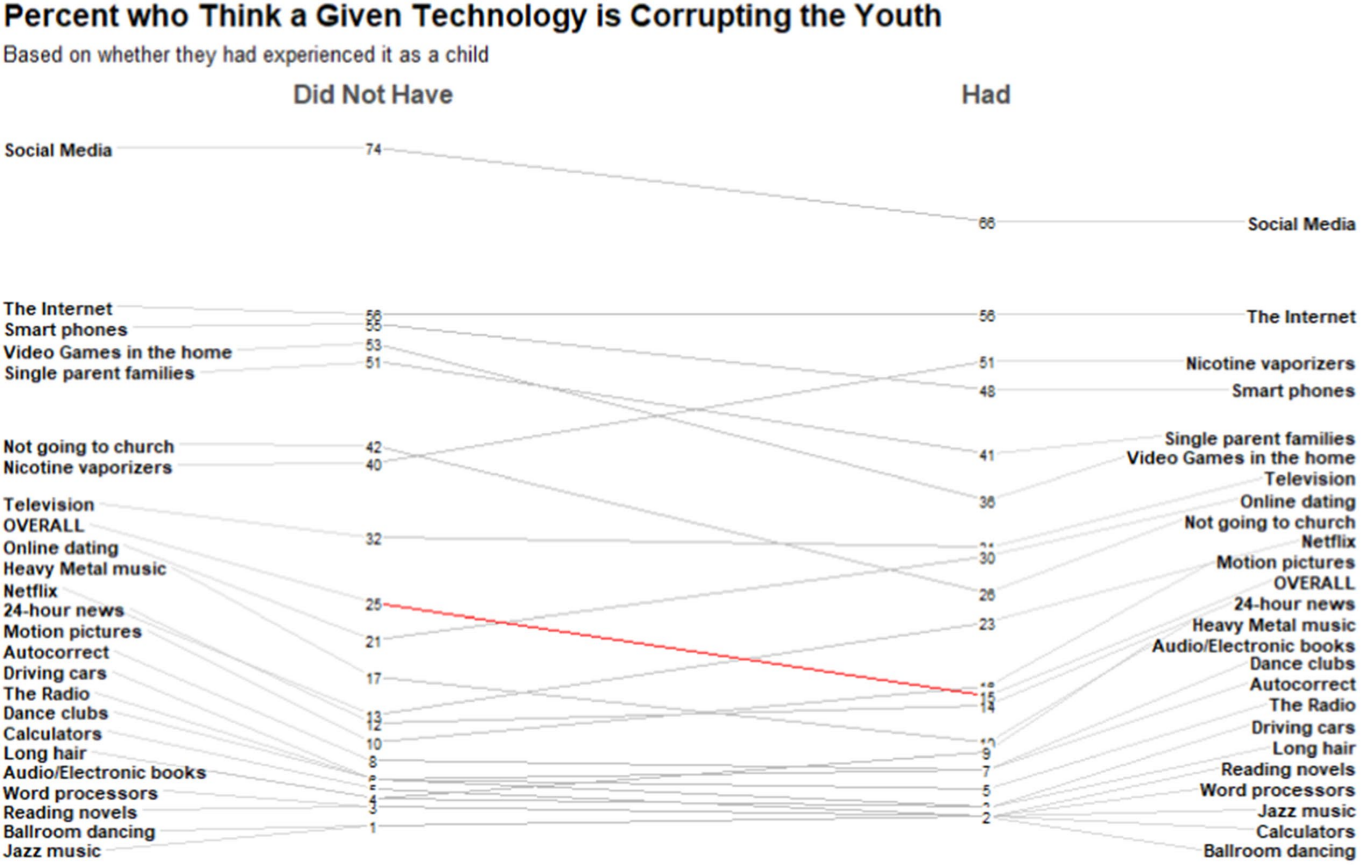
Year of Invention (or Popularization, denoted by an asterisk) and the extent to which people in general think those technology/societal advances are corrupting the youth. Line is a lowess line; gray bars are 95%CI that start once enough data becomes available.

First, as an exploratory analysis, we investigated whether there was a relationship between the year of a technology/social advance’s invention and the extent to which it is seen as corrupting. Although not preregistered, this analysis naturally arises from the conjecture that personal experience impacts perceptions of which technology/societal innovations are especially corrupting or benign. We coded each year that each technology/societal advance was invented. In three cases where there was no introduction date (i.e., Long hair; Single-parent homes; Not going to Church) so dates were set to a time seen as generally coinciding with the period in which that societal change became of note: 1969 for Long Hair to coincide with the Hippie movement; 1989 for Single-parent homes to coincide with the popular scapegoating of rising crime in the late 1980s and early 1990s in the U.S. on single-parent homes (e.g., Cohen, 2012); and 2019 for Not going to church to represent the lowest attendance rate at church in the U.S. to that point (Jones, 2021). People in general believed that more modern technologies/societal changes are more corrupting than those that had been invented or popularized earlier (*r*_S_ = 0.67, *p* < 0.001; see Figure 1). Note that removing the three items that did not have introduction dates does not alter the strength of significance of the relationship (*r*_S_ = 0.65, *p* = 0.001).

We turn now to our preregistered examination of whether people think a technology or societal advance is more corrupting if they did not experience it themselves growing up. A univariate, item-by-item, analysis could be susceptible to an age confound, whereby older participants are less likely to be exposed to a given technology growing up (e.g., Netflix) and due to their age also happen to hold more negative views of the youth (e.g., Protzko and Schooler, 2022). This confound, however, would not operate on the within-person analysis, as such general negative views (between-person effects) are statistically divorced from the within-person effect of being exposed to a given technology of societal advance growing up. Therefore, a mixed-effects analysis is able to disentangle the within-person effect (context for growing up exposed to a specific technology) from the between-person effects (random intercepts of tendency to believe in overall corrupting influences).

**Figure 2:**
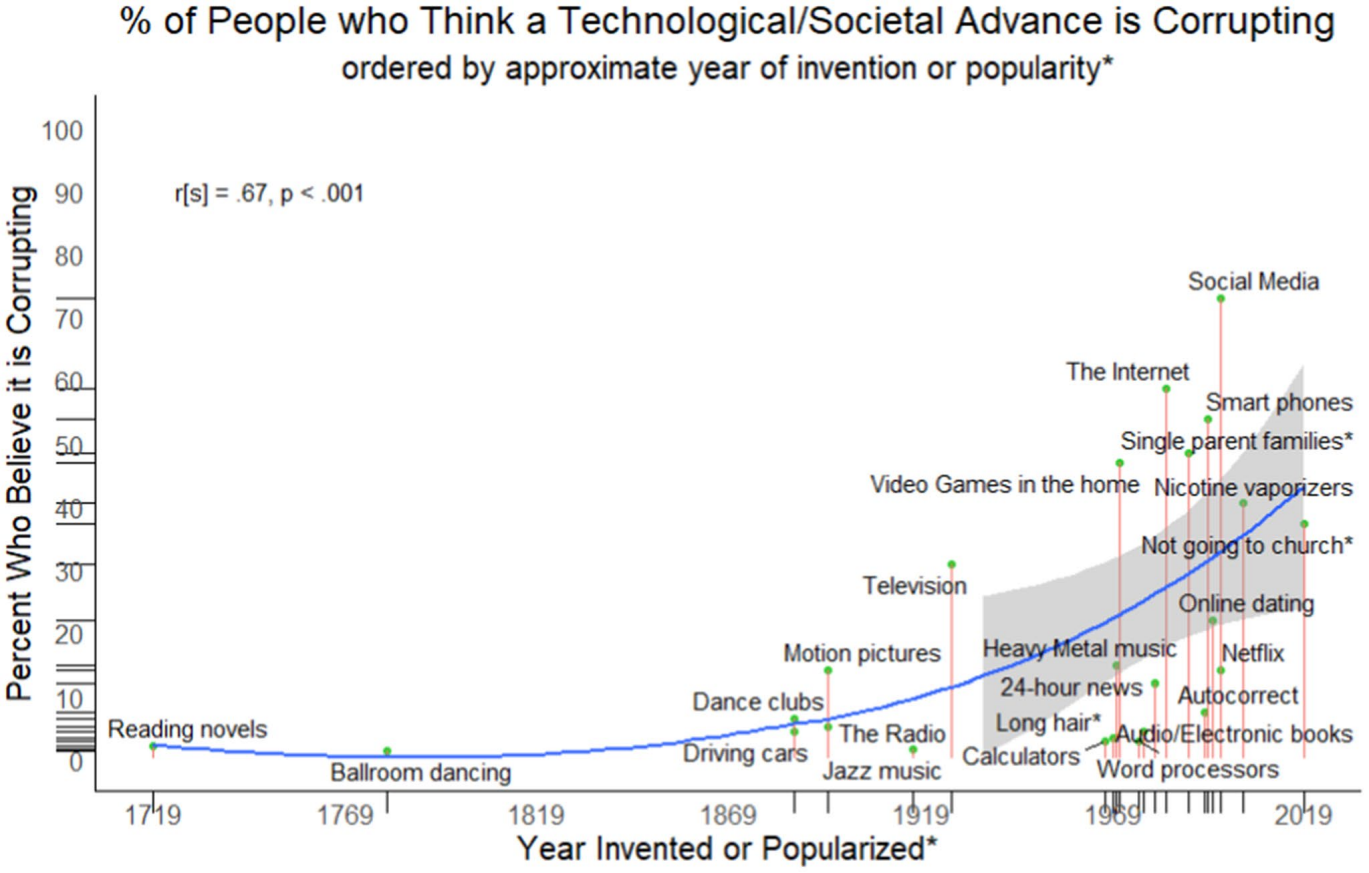
Slope graph of the percent of people who believe that a given technology or societal advance is corrupting the youth of ‘the present’, based on whether they had personal exposure to the technology or societal advance in their own childhoods. Red line is the overall model effect, people were 56% more likely to believe a given technology/societal advance was corrupting, on average, if they had not personally experienced it growing up.

Results from this overall mixed-effect model confirmed our hypothesis. If someone did not have the context of being exposed to a given technology or societal advance growing up, they were more likely to believe that it has a corrupting influence on the youth of ‘the present’ (*b* = −0.09, SE_robust_ = 0.01, *p* < 0.001, 95%CI = [−0.11, −0.09]; see Figure 2). This relationship did not change when including the item as a fixed effect in the model (*b* = −0.04, SE_robust_ = 0.01, *p* < 0.001, 95%CI = [−0.05, −0.03]). This corresponds to a 56% increase, across technological/societal changes, in the belief that something corrupts the youth of ‘the present’ if someone did not experience it themselves growing up.

We also observed that the older someone is, the more likely they were to think that technological/societal changes are corrupting (*b* = 0.001, SE_robust_ = 0.0002, *p* < 0.001, 95%CI = [0.0005, 0.001]). Nevertheless, conditioning on age (*M*_age_ = 50.1, range 18–87) did not change the results: people were still more likely to think a technological/societal changes was more corrupting if they did not experience it growing up (*b* = −0.04, SE_robust_ = 0.01, *p* < 0.001, 95%CI = [−0.05, −0.03]). Thus, overall, older participants are more likely to see corruption of the youth, but even then having not grown up experiencing a given technological/social change corresponds to an increase in the belief that it is corrupting the youth, on average.

Finally, participants were randomly assigned to fill in what they believe contributes to the corruption of the youth either first or after describing what they had access to as a child. The relationship between personal experience and beliefs of corruption was stronger when participants indicated were first asked what they were exposed to as a child (*b* = −0.05, SE_robust_ = 0.01, *p* < 0.001, 95%CI = [−0.07, −0.04]) compared to when they were first asked why they believe corrupts the youth first (*b* = −0.03, SE_robust_ = 0.01, *p* < 0.001, 95%CI = [−0.04, −0.01]). Thus, the simple act of reminding someone that they had access to a given technological/societal advance when they were young is enough to reduce the belief that that particular advance is corrupting the youth of the present.

As an exploratory analysis, we included data on the highest level of school participants had completed or the highest degree they had received. Response options were No High School Diploma (less than 12th grade), High School Graduate- high school DIPLOMA or the equivalent (For example: GED), Associate degree in college- Occupational/vocational program or Academic program, Bachelor’s degree (For example: BA, AB, BS), Master’s or Professional School degree (For example: MA, MS, MEng, MEd, MSW, MBA), Doctorate Degree (For example: PhD, EdD, MD, DDS, DVM, JD). We found that those who had a higher education level were slightly less likely to believe that technology was corrupting (*b* = −0.005, SE_robust_ = 0.001, *p* < 0.001, 95%CI = [−0.01, −0.003]). Including education as a covariate did not alter the results that those who grew up experiencing a technology found them to be less corrupting (*b* = −0.1, SE_robust_ = 0.01, *p* < 0.001, 95%CI = [−0.11, −0.09]). Indeed, even restricting the dataset to just those with the highest level of education (doctoral degrees, *n* = 63) still shows that those who experienced a given technology/societal advance growing up were less likely to believe it was corrupting (*b* = −0.11, SE_robust_ = 0.02, *p* < 0.001, 95%CI = [−0.16, −0.07]). Thus, while those who are of higher education may be less likely to believe technologies/societal advances are corrupting in general, they are just as susceptible to believing what they did not grow up with is dangerous.

## Discussion

We first confirmed that technological/societal changes that are more recent are seen as more dangerous to the youth than those of the past. So, for example, social media, the internet, and smartphones were seen as corrupting the youth of the present, while driving cars, jazz music, and dance clubs are no longer seen as dangerous. Secondly, we demonstrated that people generally believe that technologies and societal innovations they did not personally experience growing up are uniquely dangerous to today’s youth. Overall, participants who did not grow up experiencing a particular technological/social change showed a 56% increase in believing that specific aspect of life is corrupting the youth of ‘the present’ relative to participants who did grow up with it.

These findings suggest that newer technologies/societal advances are often the targets of concern for the very reason that we lack the personal experience of growing up with them. American adults held varying beliefs on the corrupting influence of the items we presented. Social Media was seen as the most corrupting influence (74%) on the youth of ‘the present’, for example, but if someone grew up experiencing social media, they found it a considerably less dangerous force (66%). The same is also said about growing up being exposed to Heavy Metal music; people who grew up not experiencing Heavy Metal music see it as particularly corrupting (17%), but much less so when they experienced heavy metal themselves (10%).

As shown in Figure 2, technologies/social changes that were invented or popularized more recently are more likely to be seen as dangerous. Our list was created from lists of historical complaints, as well as generated freely by participants. Therefore, at some point in time people believed these items to be dangerous. We confirmed that a number of technological/social changes that have historically been seen as corrupting or dangerous to the youth when they first appeared are no longer thought to be dangerous. In 1843, for example, warnings were raised about the dangers of ballroom dancing to the youth: “If you wish to preserve in its freshness their modest innocence…suffer them not to waltz” (The Waltz, 1843, p. 152); yet in 2019, only 1% of American adults saw ballroom dancing as a corrupting influence on the youth of ‘the present’. Thus, there is considerable heterogeneity across what people believe is corrupting, both presently and historically.

Why do we keep believing each new technological/social change comes with it a danger to the youth? Here we start to explore one reason, lack of personal experience. Someone growing up in a single parent house has context for what it is like, someone who grew up in a dual parent house does not, for example. Presumably, when someone has personal experience with a technology/societal advance they may see it is as less problematic than they might have otherwise thought. They also may be more reluctant to express concern about growing up with a technology/innovation that might bear on their own proficiencies.

As people get older, they are more critical of the youth of ‘the present’ (Protzko and Schooler, 2022). We also show here that as people age, they tend to see more technological/societal changes as corrupting the youth overall. This may be because as people age their memories for their childhood become more favorable (Field, 1997) and they view the past as more idyllic (Eibach et al., 2003 Protzko and Schooler, 2019; see also Mastroianni and Gilbert, 2023). Thus, not only do youth of ‘the present’ seem in decline as people age, but society seems in decline; older people are more likely to search for explanations involving technologies/societal changes they do not have context growing up with.

People are similarly critical of technology that specifically was invented after they were born (Smiley and Fisher, 2022). Our work here connects to this literature and helps explain why. Technology invented while one is alive is potentially less likely to have been experienced directly while growing up, especially if that technology was invented in one’s adulthood. This may be why so many older adults simultaneously have social media accounts yet believe those social media sites are dangerous for children. A lack of context for social media while growing up makes the societal advance seem dangerous.

We also found an important mitigation strategy, simply reminding people of what technologies/societal advances they grew up with is associated with a decreased belief in the corrupting influence, strengthening the bond between experience and belief. This presents the intriguing possibility that simply being reminded of what one was actually exposed to as a child may reduce the belief that those technologies or societal changes are corrupting. Similar research has explored the idea that such simple reminders can reduce beliefs in the decline of society; asking people to reflect on how their own driving ability has changed in the past 10 years reduces the belief that other people’s driving ability has become more aggressive, reckless, for example (see Eibach and Libby, 2009; see also our replications at https://osf.io/xrbfp/). Our results may extend future work about the power of simple contextual reminders on reducing prejudice against the youth and panics about the next technological/societal change.

### Implications

Of course, the fact that personal experience with a societal innovation reduces people’s concern about its impact should not necessarily allay fears about any particular new technology or societal advance. Similarly, just because people are generally less uneasy about older innovations relative to newer ones does rule out the possible emergence of a truly dangerous new innovation. Nevertheless, fostering a general awareness of the present findings may help to contextualize the concern that current technologies are harming today’s youth. Remembering that adults were similarly distressed about the technologies that we used as youth, and understanding that part of the reason we see new technologies and societal changes as corrupting of the youth of ‘the present’ is that we personally lacked exposure to them growing up, could temper our fears. More generally, in debates, both present and future, about the dangers of new technologies and societal advances, the work here can be invoked to argue that the worries and intuitive sense of danger we will continue to fear are not necessarily accurate. Those fears may be influenced by a simple lack of exposure to the new advance.

Concerns about the corrupting impact of new technologies might also be dampened by reducing people’s ill-founded distress about the youth of the day. As noted Protzko and Schooler (2019) demonstrated that people routinely denigrate the youth of the day for reasons unrelated to the characteristics of the population at large, but rather due to qualities of themselves. Essentially people assume the youth of today are lacking in whatever particular qualities they themselves excel. Reducing our tendency to view the youth of the present as deficient may help to alleviate some of our concerns about the negative impact of new technology or social change.

### Limitations and future directions

The present findings are wholly consistent with the view that whether one grew up with a particular technology/societal change impacts their belief in its corruptive impact of the youth of ‘the present’. It must be acknowledged, however, that this observed relationship was largely correlational. It is not random who takes up a new technology or societal advance. People who are more interested in the technology or who are more receptive to the societal change are undoubtedly more likely to be early adopters. Nevertheless, we did find an effect of whether or not people were first asked to consider their own personal experience with an innovation on their assessment of its impact. The mitigating effect of remembering that one personally used a technology when growing up further helps to build a causal case that a lack of personal experience with an innovation contributes to its perceived danger.

Future work can look for exogeneous exposure of new technologies (e.g., rollouts in certain markets but not others) to further test the causal implications of these findings. It should be the case that if one is randomly exposed to a new technology growing up (because one is in a test area where the technology is available) that this will lead to less concern about it relative to those who were not exposed to it. Furthermore, future work can explore the causal effect of presenting the findings here on tempering fears. Experiments could test whether alerting people to the relationship between increased exposure and reduced fear can cause people to be less concerned about the new technology or social change under debate.

There may also be a concern about the prompt used in the main study where participatns were told: “Children and youths today appear noticeably in decline from the standards of youth of the past. We would like to know what you think the reasons or causes of this decline are.” before being asked what they believed the cause of any declines are. We believe this is not an issue of a demand effect for a number of reasons. First, demand effects have been shown to not reliably exist in research conducted with non-student samples (for a meta-analysis, see: Coles and Frank, 2023). Second, the belief that the youth are in decline is pervasive and has been demonstrated in numerous studies (e.g., Trzesniewski and Donnellan, 2014; Protzko and Schooler, 2019 2022), and prompting would likely not induce such a belief. Third and most importantly, this would not alter the within-person relationship between not experiencing a given technology and thinking that technology is particularly dangerous. Still, future research, especially if using student samples, should be aware of such possible demand effects.

## Conclusion

We can never know what it is like to grow up any other way than we did. We only have our own experience, no more and no less. This experience apparently matters for later beliefs about society. We are more likely to see a given technology or societal change as a corrupting, damaging force on the youth of ‘the present’ if we did not have the context for what it was like to experience it growing up. As society changes more rapidly and technological innovations become more frequent (e.g., Kurzweil, 2004), we will continue to find ourselves in a world that looks different from the one we grew up in—seeing danger for the next youth. This apparent recurring process—of innovations being spurned as corrupting by older generations who did not grow up with them—could continue in perpetuity. Considering one’s own experience with a technology, however, serves to mitigate projections of its negative impact on the young, potentially suggests one way to escape this perennial cycle. If older generations remember that they thrived in the context of the novel technologies and societal advances of their day, they may gain a more optimistic vision that current youth can similarly prosper despite or perhaps even because of the new developments that they grow up with.

## Data availability statement

The datasets presented in this study can be found in online repositories. The names of the repository/repositories and accession number(s) can be found at: https://osf.io/4zk9e/.

## Ethics statement

This study was found exempt by the University of California, Santa Barbara Office of Research and Human Subjects (IRB).

## Author contributions

JP came up with the idea, programmed the study, and collected and analyzed the data. All authors refined the methods, wrote the original draft, and revised the manuscript.

## Funding

The research was funded by the John E. Fetzer Memorial Trust.

## Conflict of interest

The authors declare that the research was conducted in the absence of any commercial or financial relationships that could be construed as a potential conflict of interest.

## Publisher’s note

All claims expressed in this article are solely those of the authors and do not necessarily represent those of their affiliated organizations, or those of the publisher, the editors and the reviewers. Any product that may be evaluated in this article, or claim that may be made by its manufacturer, is not guaranteed or endorsed by the publisher.
